# Repeatability, reproducibility, and the effects of radiotherapy on radiomic features of lowfield MR-LINAC images of the prostate

**DOI:** 10.3389/fonc.2024.1408752

**Published:** 2025-01-20

**Authors:** Parker Anderson, Nesrin Dogan, John Chetley Ford, Kyle Padgett, Garrett Simpson, Radka Stoyanova, Matthew Charles Abramowitz, Alan Dal Pra, Rodrigo Delgadillo

**Affiliations:** Department of Radiation Oncology, University of Miami Miller School of Medicine, Miami, FL, United States

**Keywords:** radiomics, prostate, repeatability, reproducibility, T2, MRI, MR-LINAC, ViewRay

## Abstract

Definitive radiotherapy (RT) has been shown to be a successful method of treating prostate cancer (PCa) patients. Through radiomics, a quantitative analysis of medical images, it is possible to adapt treatment early on, which may prevent or mitigate future adverse events. During RT of PCa, low-field magnetic resonance (MR) images, taken with a LINAC onboard imaging system in a process known as magnetic resonance-guided radiotherapy (MRgRT), are used to improve treatment accuracy via superior setup compared to x-ray methods. This work investigated baseline repeatability of radiomic features (RFs) by comparing planning MR images (pMR) with first-fraction setup images (FX1) taken with onboard MRI. The changes in RFs following RT were also looked at with the use of last-fraction setup images (FX5). Earlier research has investigated the use of planning images from cone beam CT (CBCT), but to our knowledge no research has previously shown the relationship with onboard MRI. The correlation between FX1 images and 3T diagnostic MR (dT2) images was also studied. Forty-three first and second order radiomic features extracted from these images were compared by calculating Lin’s concordance correlation coefficient (with Benjamini-Hochberg correction for multiple comparisons) between the modalities. FX1 and pMR images were correlated (p<0.05) for all but one RF. 12 RFs correlated between pMR and dT2 images. There was a noticeable change in correlation values for RFs when looking at FX1 and FX5 images, with only 15 correlating significantly. The change in correlation values between pMR and FX5 images was comparable to that between FX1 and FX5 images, with 33 features having a CCC value deviation of less than 0.1. These results demonstrate that RF features are repeatable across different images of the same modality without treatment intervention. This study has also shown a noticeable, reproducible change in RFs as RT goes on. Reproducibility of RFs between different modalities was not strong. This study demonstrated that we can reliably use onboard MRI to observe day-to-day feature changes as a result of RT.

## 1 Introduction

Prostate cancer (PCa) is the most common malignancy among men and the second-leading cause of cancer-related mortality in the United States (US) (1). With 3.1 million PCa survivors in the US, acute and late side effects of PCa treatment impact a sizable proportion of US men (2, 3). Definitive radiotherapy (RT), a primary intervention for intermediate and high-risk PCa, aims to limit treatment-related side effects and preserve patient quality of life while delivering curative dose to the prostate (4–7). Magnetic resonance-guided RT (MRgRT) with onboard MRI can improve treatment outcomes via enhanced visualization of the prostate, allowing for reduction of planning target volume (PTV) margins and increased sparing of adjacent organs at risk (OAR) (8). However, despite the many modern innovations in RT techniques and technologies, PCa treatment outcomes can still entail adverse effects and incomplete treatment of the target volume.

Radiomics, a field of study involving the extraction of quantitative characteristics from medical imaging, has been previously integrated into models predicting outcomes of PCa treatment (9–15), often successfully. Delta-radiomics takes this field of study a step further; radiomic analysis is applied at multiple points throughout treatment to identify tumor changes, often specifically due to RT intervention (16). There is increasing evidence in support of using delta-radiomic features extracted from MR imaging as non-invasive biomarkers to predict PCa treatment outcomes. A recent study has shown RFs extracted on a low-field magnet to be repeatable in both a phantom and between patient scans taken shortly after one another on the same day (17), but to our knowledge no one has analyzed the repeatability of RFs from scan-to-scan in actual patients over a longer period of time. Longer time between scans can result in increased variability in patient anatomy, which may have an effect on RFs. If the features do not correlate between images taken on the same modality at various times, then it is unclear if delta-radiomic analysis is valid in future studies. Some studies have shown that RFs can be dependent on the pulse sequences (18) and acquisition and reconstruction methods (19) used to acquire them, demonstrating that they are somewhat dependent on imaging modality.

Previous studies in this area have compared the use of cone-beam CT (CBCT) and MRI (13), but none have investigated the correlation of radiomic features (RFs) between planning MR (pMR) images and the daily setup MR images taken prior to treatment (FX1). We also wanted to see how RFs were affected as treatment progressed by comparing FX1 and pMR images with those taken prior to the last fraction of treatment (FX5). In this study, we hypothesized that there would be strong repeatability between features of the same imaging modality, specifically between pMR and FX1 images since they are images taken of the same target volume on the same machine, both prior to RT intervention. We predicted that there would be some noticeable change in RF correlation between FX1 and FX5 images, as the prostate volume has been almost completely treated in the time between when these two images were taken. It was postulated that this observed change could be reproducible, so the relationship between pMR and FX5 images was also investigated. We also hypothesized that there would be reasonable correlation between pMR and 3T diagnostic MR (dT2) images, however not as strong as between pMR and FX1.

Recent delta-radiomic efforts involving the prostate have proven to be especially useful in obtaining predictive data for PCa treatment outcomes (20). Unlike the traditional diagnostic MRI imaging, onboard MR images are obtained with low-field MRI that is onboard an MR-LINAC system (such as ViewRay, Mountain View, CA, and Elekta Unity MR-Linac, Elekta AB, Stockholm, Sweden). These images are used to ensure quality of daily treatment and can be used to observe day-to-day changes in RFs. The purpose of this study was to determine the correlation of prostate RFs between FX1 and pMR images, FX1 and FX5 images, pMR and FX5 images, and FX1 and dT2 images. Determining the correlations between RFs of each of these modalities may allow us to have a better understanding of radiomic data acquisition of MR for PCa and characterize the efficacy of future MR-LINAC delta-radiomic studies.

## 2 Methods

### 2.1 Patient population

Twenty patients enrolled in institutional review board (IRB)-approved treatment protocols for PCa were selected for this study. The ethical approval for this study was obtained from the University of Miami Institutional Review Board (IRB). Data from each patient was selected and analyzed retrospectively, and all methods and analysis done in this study were conducted under proper guidelines and regulations.

All patients were being treated for malignant neoplasm of the prostate with varying dose schemes, all with curative intention. Four patients were treated in forty fractions at 2 Gy per fraction, totaling to 80 Gy. The sixteen other patients were treated in five fractions at 8 Gy per fraction, totaling 40 Gy. Every patient was treated on an MR-LINAC (ViewRay, Mountain View, CA) that is equipped with an onboard MRI guidance system.

### 2.2 MR-LINAC

The MR-LINAC that was used possesses a 6MV beam and is a machine through which real-time MR images of the patient can be taken during the treatment process with use of a 0.35T magnet (ViewRay, Mountain View, CA). The implementation of this machine may be beneficial in delta-radiomic studies due to the daily images taken as treatment progresses. During treatment, the pulse sequence this device uses is a balanced steady-state free precession sequence, which is a type of balanced sequence yielding a hybrid T2/T1 contrast, slightly weighted toward T2 (21). Because of this, images taken on the MR-LINAC are compared to diagnostic T2 images in this study.

### 2.3 Imaging

FX1 images were used as the baseline for each comparison. FX1 can be defined as the image used for setup prior to the first fraction of RT, taken on the 0.35T MR-LINAC. FX5 is the setup image taken prior to the administration of the last fraction of treatment. pMR images were also taken on the MR-LINAC prior to treatment, and were the original images used for treatment planning. FX1 images were also compared to dT2 images, which are those used for original diagnosis of disease and were taken on 3-Tesla diagnostic scanners (see **Table 1**) utilizing various T2 protocols.

**Table 1:** Machinery used to produce dT2 images used in this study.

Manufacturer	Model	Number of patients
GE Medical Systems	DISCOVERY MR750	4
GE Medical Systems	OPTIMA MR360	1
Philips Medical Systems	Achieva	1
Siemens	Avanto	2
Siemens	MAGNETOM Vida	2
Siemens	Skyra	3
Siemens	TrioTim	3
Siemens	Verio	4

Patient cohorts of each comparison were different in order to increase the robustness of our analysis. All 20 patients were included in the comparison between FX1 and pMR. Fourteen out of the twenty patients were included in the comparison between FX1 and dT2, since not all patients had their diagnostic imaging acquired on the same type of machine. We narrowed the cohort of this comparison to only include patients imaged on Siemens brand scanners, as there is less variability in acquisition parameters (pulse sequence, TE, TR, etc.) within this group than when including the other machines. Sixteen patients were included in the comparisons with FX5 images, all of which had fractionation schemes of 40 Gy in five fractions. We wanted to ensure that the amount of RT intervention between FX5 and the other scans remained the same for each patient in this comparison, so the four patients that were not included were those that had fractionation schemes of 80 Gy in 40 fractions.

### 2.4 Contouring

The prostate served as the region of interest (ROI) for the RF extraction. The contouring of each image was performed by a team of researchers with expertise in PCa and delineation, consisting of radiation oncologists and physicists. First, prostate contours from pMR images were transferred to other studied images (dT2, FX1, FX5) using a rigid registration with the assistance of a radiation oncology imaging software (MIM, ver. 6.8.1, MIM Software Inc., Cleveland, OH). Then, corrections were made to each transferred contour to ensure that ROI volume was as similar as possible for each image and anatomically correct.

### 2.5 Description of radiomic features

Forty-three RFs were extracted from prostate contours on diagnostic T2 and pMR images. These specific forty-three were used as they are from the most commonly studied RF classes. RFs from these classes have demonstrated predictive relationships with useful prostate cancer metrics, such as aggressiveness (15), progression (20), and identification and segmentation of disease (22). Though more RFs appear in other works, it has been documented that many RFs are intercorrelated and thus not specifically unique from one another (22–24). Therefore, this study focused on the most typically studied RFs. Prostate volume was also included as a descriptor to serve as a control and to ensure that regions investigated were constant across modalities. RFs were calculated using the MATLAB (MATLAB, ver. R2022a, Math-Works Inc., Natick, MA) “Radiomics” package developed by Vallieres, et al. in combination with in-house code to extract 3D bitmaps of the ROI using the DICOM structure files from the MRI DICOM files (25). RFs from five classes were extracted: first-order statistical features (Global), Gray-Level Co-Occurrence Matrices (GLCM), Neighborhood Gray-Tone Difference Matrix (NGTDM), Gray-Level Run Length Matrices (GLRLM), and Gray-Level Size Zone Matrices (GLSZM). These features comply with the Image Biomarker Standardization Initiative (IBSI). For IBSI codes of features mentioned, refer to **Table 2**.

**Table 2:** List of RFs used for study.

Feature Class [IBSI code]	Features [IBSI code]
Gray-Level Co-Occurrence Matrices (GLCM)	Contrast [ACUI]
[LFYI]	Correlation [NI2N]
	Dissimilarity [8S9J]
	Energy [8ZQL]
	Entropy [TU9B]
	Homogeneity [IB1Z]
	Sum Average [ZGXS]
	Variance [UR99]
Gray-level Run Length Matrices (GLRLM) [TP0I]	Gray-level Non-Uniformity (GLN) [R5YN]
	Gray-level Variance (GLV) [8CE5]
	High Gray-level Run Emphasis (HGRE) [G3QZ]
	Low Gray-level Run Emphasis (LGRE) [V3SW]
	Long Run Emphasis (LRE) [W4KF]
	Long Run High Gray-level Emphasis (LRHGE) [3KUM]
	Long Run Low Gray-level Emphasis (LRLGE) [IVPO]
	Run-Length Non-Uniformity (RLN) [W92Y]
	Run Length Variance (RLV) [SXLW]
	Run Percentage (RP) [9ZK5]
	Short Run Emphasis (SRE) [220V]
	Short Run High Gray-level Emphasis (SRHGE) [GD3A]
	Short Run Low Gray-level Emphasis (SRLGE) [HTZT]
Gray-level Zone Size Matrices (GLZSM) [9SAK]	Gray-level Non-Uniformity (GLN) [JNSA]
	Gray-level Variance (GLV) [BYLV]
	High Gray-level Zone Emphasis (HGZE) [5GN9]
	Low Gray-level Zone Emphasis (LGZE) [XMSY]
	Large Zone Emphasis (LZE) [48P8]
	Large Zones High Gray-level Emphasis (LZHGE) [J17V]
	Large Zones Low Gray-level Emphasis (LZLGE) [YH51]
	Short Zone Emphasis (SZE) [5QRC]
	Short Zones High Gray-level Emphasis (SZHGE) [HW1V]
	Short Zones Low Gray-level Emphasis (SZLGE) [5RAI]
	Zone Percentage (ZP) [P30P]
	Zone Size Non-Uniformity (ZSN) [4JP3]
	Zone Size Variance (ZSV) [3NSA]
Neighborhood Gray-Tone Difference Matrix (NGTDM) [IPET]	Busyness (BUSY) [NQ30]
	Coarseness (COAR)*
	Complexity (CPLX) [HDEZ]
	Contrast (CONT) [65HE]
	Strength (STRG)*
Intensity-based Statistics (Global) [UHIW]	Kurtosis [IPH6]
	Skewness [KE2A]
	Variance [ECT3]

RFs denoted with (*) are calculated as defined by Amadasun and King (36).

IBSI defines image processing as including procedures such as interpolation, range-re-segmentation, quantization, and image filtering (26). Quantization algorithm can be defined as how intensities of an image are quantized into 64 discrete bins. Previous work by Delgadillo et al. (27) demonstrated that image processing with use of the Lloyd-Max quantization algorithm with Collewet normalization (Llo-1) led to the best balance of repeatability and reproducibility in extracted RFs, so this is the processing that was used prior to extraction. Collewet normalization is a normalization where the gray levels of the ROI are normalized from the range of [µ_R_−3σ_R_, µ_R_ +3σ_R_] where µ_R_ was the mean and σ_R_ was the standard deviation of the ROI gray levels (28). Lloyd-Max quantization allows for minimization of quantization error based on the way it assigns bins (29).

Other researchers have shown that some RFs may highly correlate with volume, thus making them dependent on the volume of the ROI and possibly leading to confounding data in our analysis (30, 31). To account for this, volume normalization (VN) was performed on RFs with known methods, including NGTDM Busyness, NGTDM Coarseness (30), NGTDM Strength, LSZM GLN, GLRLM GLN, and GLRLM RLN (31). GLSZM ZSN was normalized by dividing by the number of pixels using a similar logic as GLRLM RLN. Analysis was performed on these RFs after normalization of volume to reduce their values’ dependence on prostate volume, ensuring a robust comparison. For some other RFs that appear to be volume dependent, no known VN was found in the literature.

### 2.6 Data analysis

**Figure 1:**
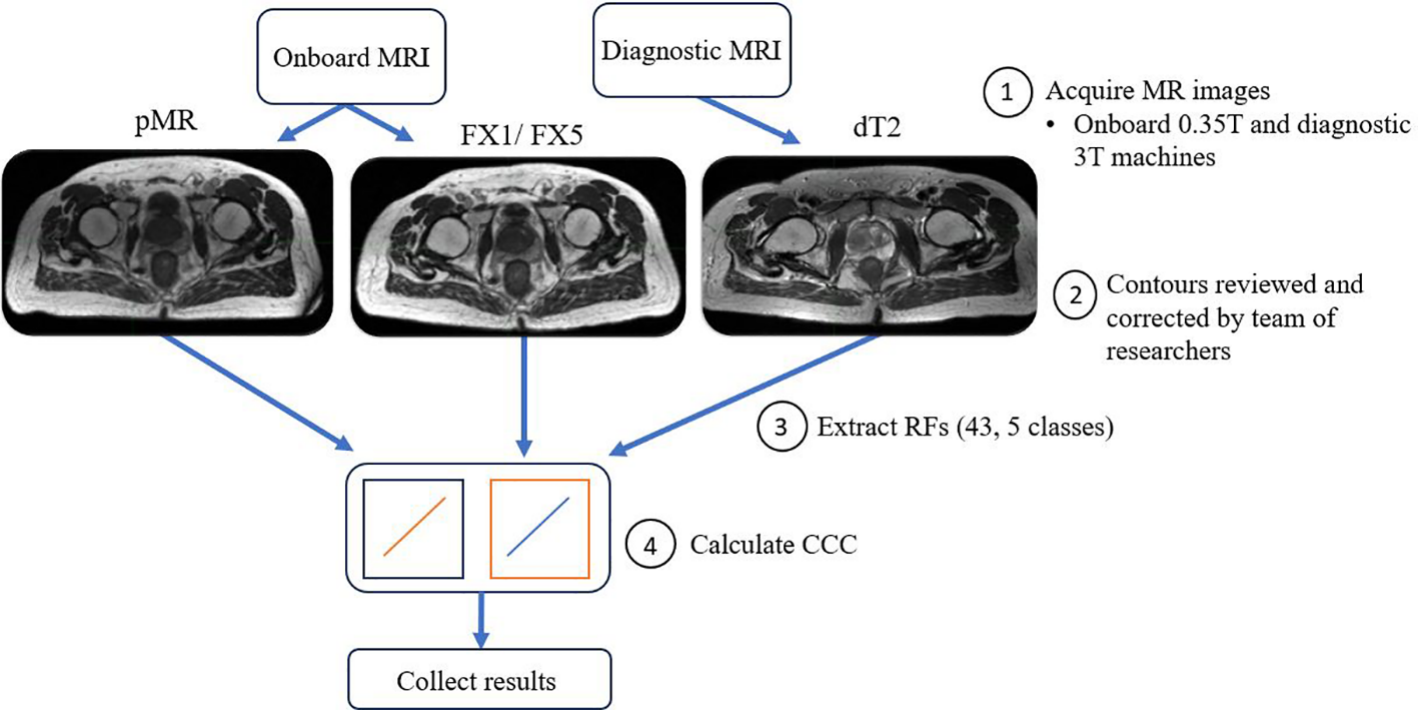
Schematic picture of overall workflow of this study.

The main aim of our analysis was to see how well RFs correlated between different images of the same patient treated on the MR-LINAC. To do this, Lin’s concordance correlation coefficient (CCC) was calculated in MATLAB between FX1 images and the other imaging modalities. CCC was used as we wanted to assess the deviation from perfect agreement between the different sets of images, i.e. measure how well our test (FX1) and retest (pMR) images correlated (32). CCC has been used as a standard of comparison for several previous radiomics reproducibility studies (27, 33). These calculations allow us to measure the strength in relationship between features across fractions and see how they deviate from a fixed reference point, i.e. FX1 images. For visual representation of the workflow of this analysis, refer to **Figure 1**. Statistical significance of these correlations was determined by calculating p-values via Student’s t-test. Benjamini-Hochberg correction was also applied to this correlation’s p-value, which decreases the likelihood of false discovery of significance. A precedence for using the Benjamini-Hochberg for multiple comparison was found in a lung-based radiomics paper by Fave et al., 2016 (30). This allowed for the reduction of false positive correlations and accounts for multiple comparisons. This process was repeated between FX1 and pMR images, FX1 and FX5 images, pMR and FX5 images, and FX1 and dT2 images.

## 3 Results

**Figure 2:**
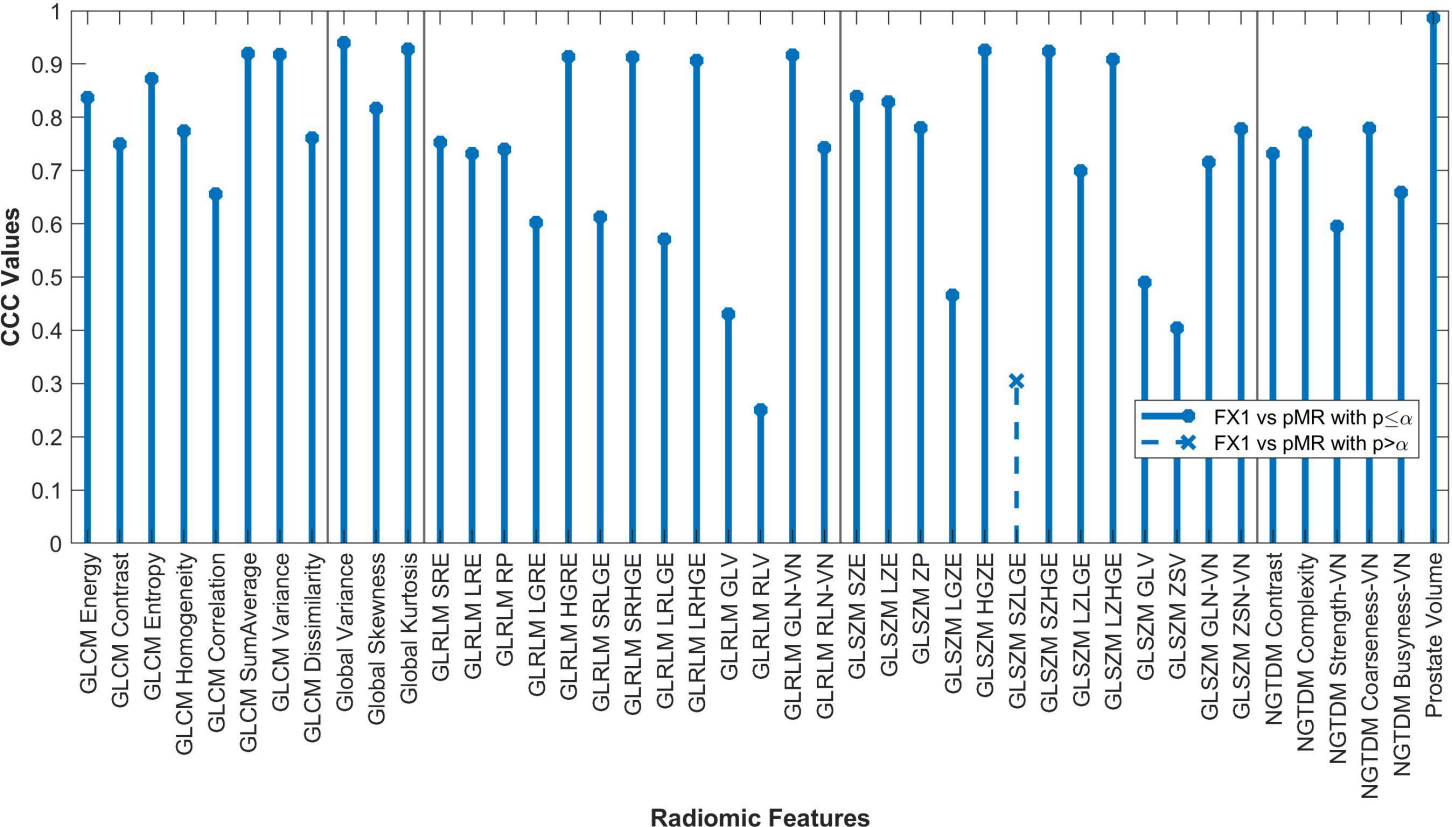
Average CCC values between FX1 and pMR images (n=20 patients) are plotted for each studied RF (classes separated by vertical line).

**Figure 3:**
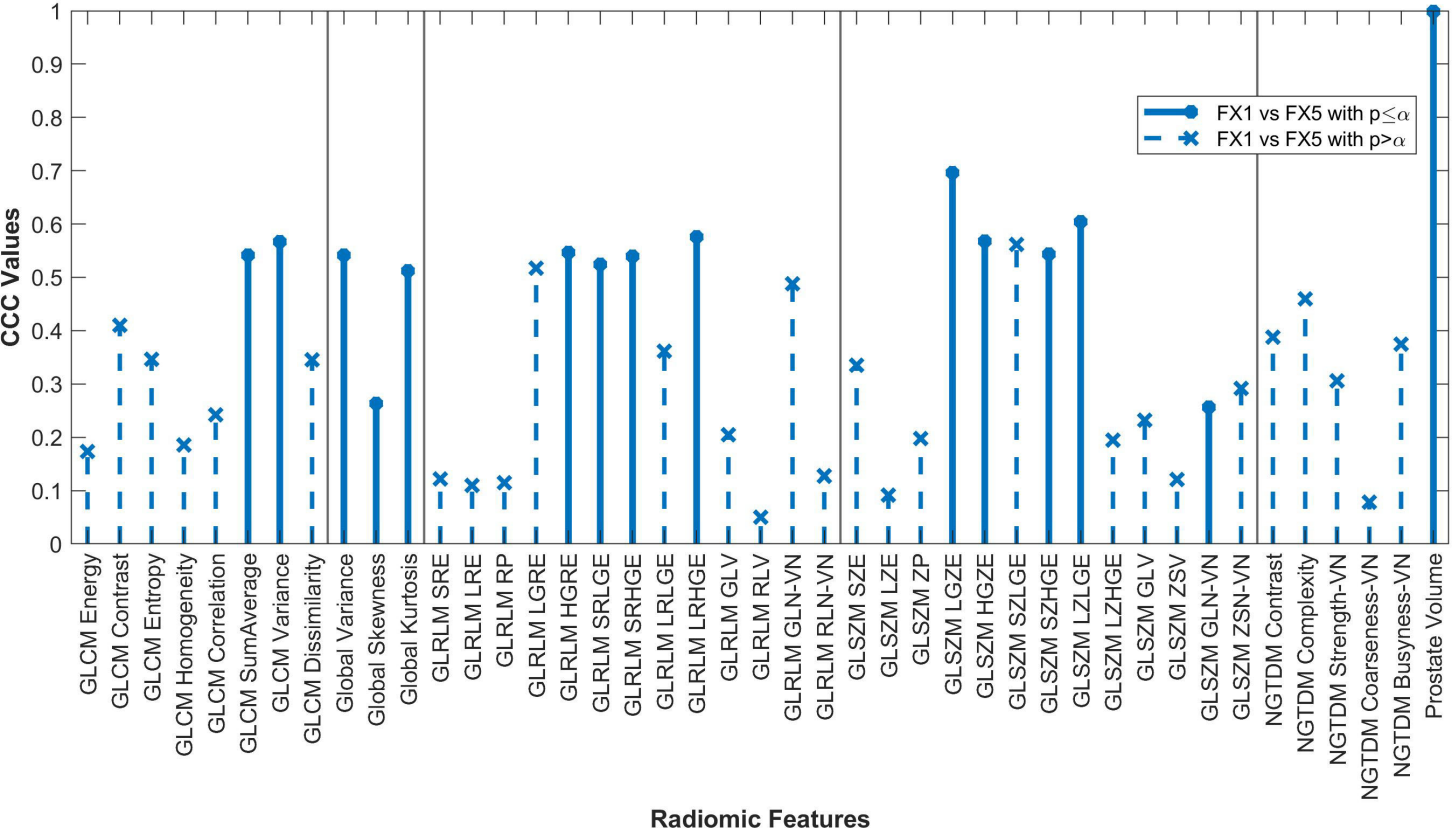
Average CCC values between FX1 and FX5 images (n=16 patients) are plotted for each studied RF (classes separated by vertical line).

**Figure 4:**
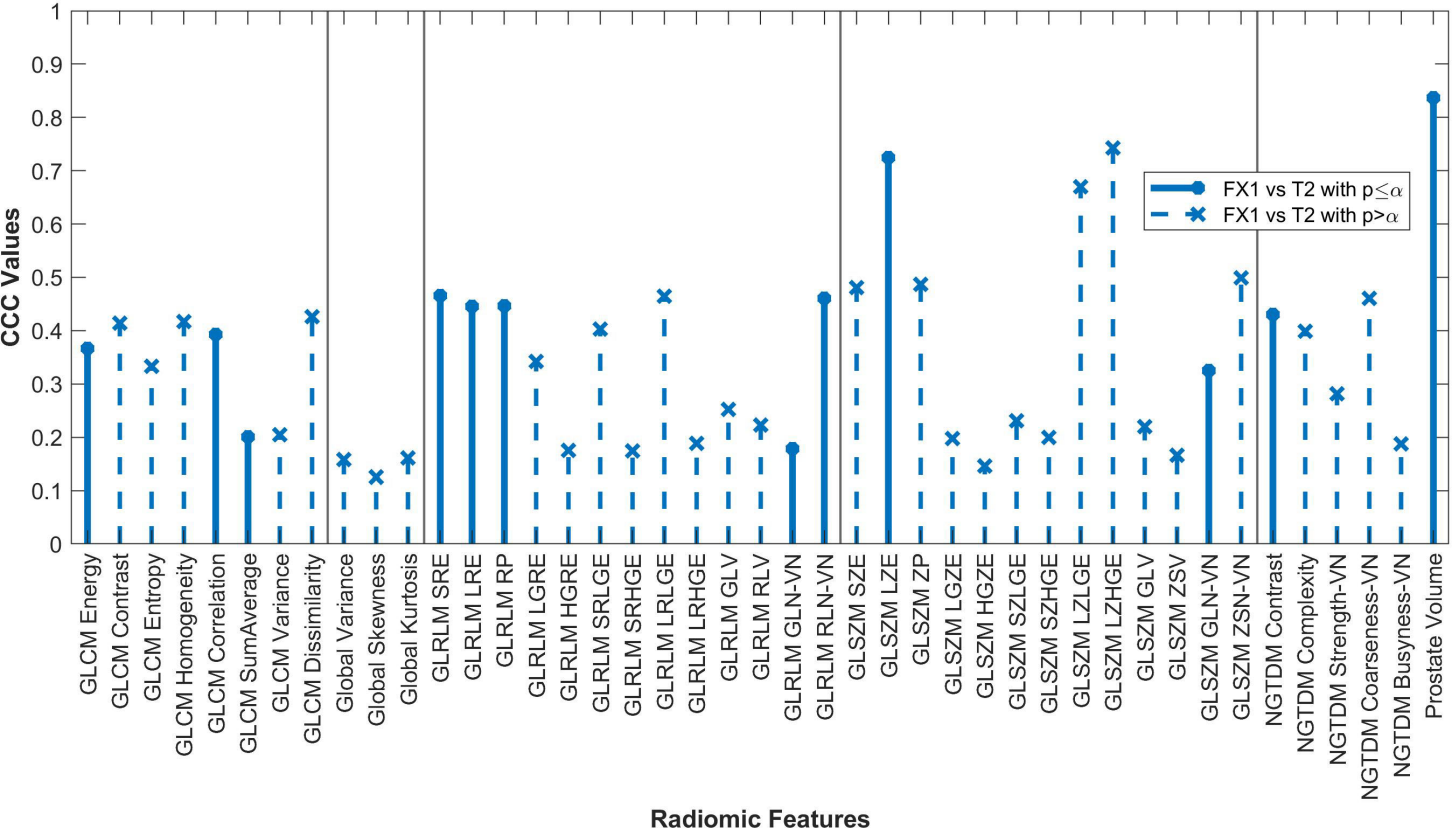
Average CCC values between FX1 and dT2 images (n=14 patients) are plotted for each studied RF (classes separated by vertical line).

Forty-two of the forty-three RFs examined significantly correlated (p<0.05) between FX1 and pMR images. 15 RFs correlated between FX1 and FX5 images. 12 RFs correlated between FX1 and dT2 images. 11 RFs correlated between pMR and FX5 images. Specific CCC values for each feature examined are shown in **Figures 2**–**4**.

By looking at plotted values of RFs between modalities, it is evident that there is a much stronger correlation between pMR and FX1 images. A clear positive correlation can be seen between these modalities, with 17 RFs exhibiting a CCC value greater than 0.80. This is likely due to the fact that these two images are taken on the same machine with the same magnet strength.

**Figure 5:**
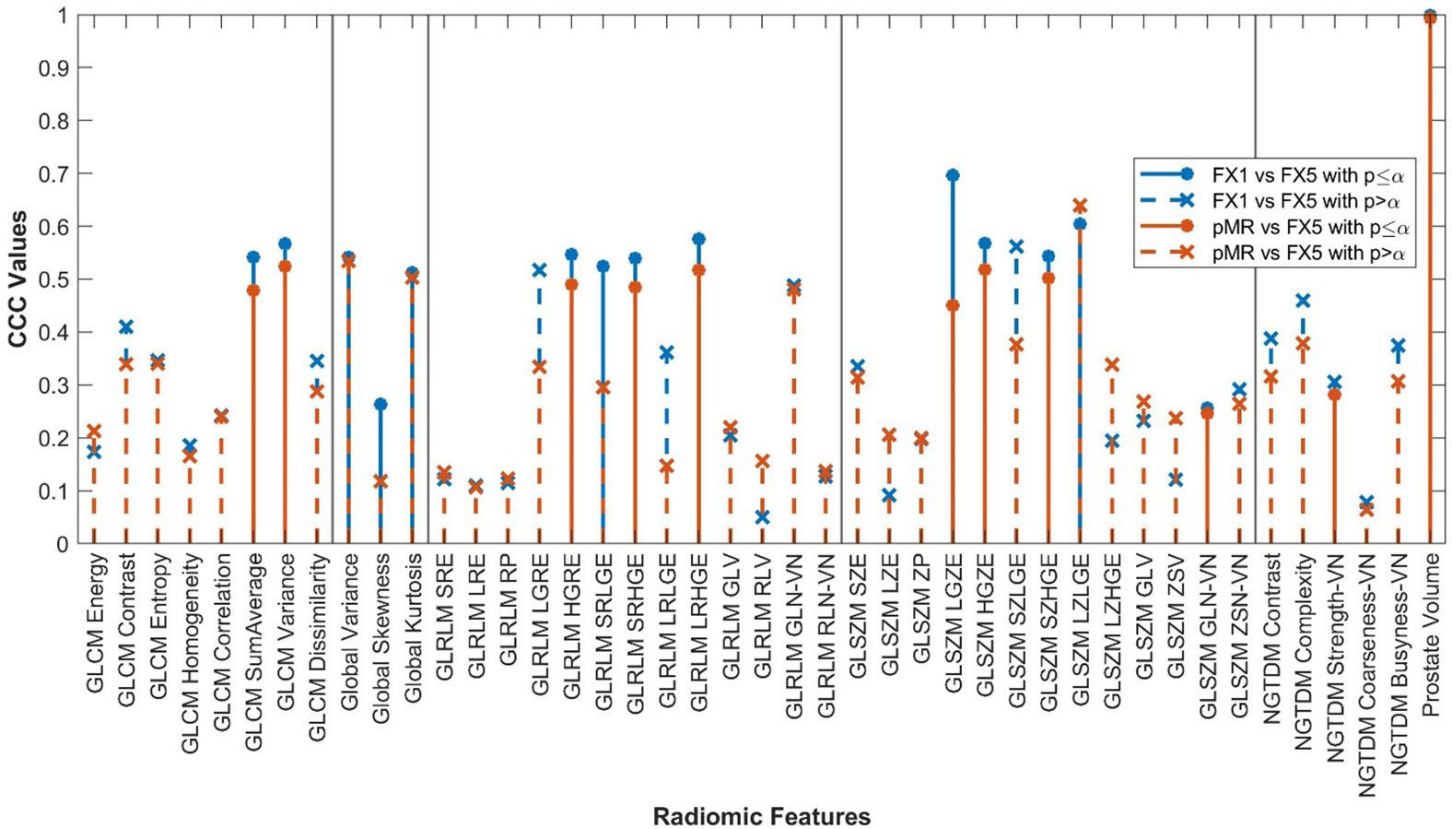
RF correlations between FX1 and FX5 images plotted against those between pMR and FX5 images. RF classes separated by vertical lines.

The changes in RFs between FX1 and FX5 images were very comparable to the changes between pMR and FX5 images. 33 RFs had a variability of less than ≤0.1 in their CCC values between these two comparisons. Refer to **Figure 5** for a complete comparison.

Imaging characteristics were calculated for images of each modality that were investigated, such as FOV, pixel spacing, and image size. These values were the same between pMR, FX1, and FX5 images, as the pMR images are used as reference for setup images. Refer to **Table 3** for a complete overview of these statistics.

**Table 3:** Statistics for imaging characteristics of each modality investigated (average ± standard deviation).

Modality	Number of patients	Image Size (pixels)	Pixel Spacing (mm)	Slice Thickness (mm)	FOV (mm)
dT2	14	421.71 ± 99.30 x 378.29 ± 96.16	0.78 ± 0.33	2.86 ± 0.40	309 ± 78.44
pMR	20	314.3 ± 30.23 x 293.5 ± 15.45	1.5 ± 0.00	1.58 ± 0.33	470.75 ± 44.89
FX1	20	314.3 ± 30.23 x 293.5 ± 15.45	1.5 ± 0.00	1.58 ± 0.33	470.75 ± 44.89
FX5	16	314.3 ± 30.23 x 293.5 ± 15.45	1.5 ± 0.00	1.58 ± 0.33	470.75 ± 44.89

## 4 Discussion

Our original hypotheses were supported by the results found in this study. RFs extracted from pMR images correlated very well with FX1 images, with all but one significantly correlating. Given that FX1 and pMR images are taken on the same machine, it is logical that images of the same target volume are similar in radiomic data. Although this may seem obvious, this analysis has demonstrated that RF data between images of the same subject taken days apart are still repeatable despite known variability in MR imaging. Since FX1 and pMR are both taken prior to treatment, there should not be much change in data between the two images. This study confirms this theory. In future analysis, we can now postulate that if there is some noticeable deviation between RF data on imaging from different fractions of treatment, it is likely due to RT intervention.

Only twelve RFs significantly correlated between pMR and dT2 images. Even though these images are of the same target volume and are both taken pre-treatment, the different imaging modalities result in too large of a difference for RFs to be consistently reproducible. The difference in magnet strength used to take each image is drastic, therefore leading to heightened differences in image quality and thus differences in the radiomic data acquired.

Fifteen RFs correlated significantly between FX1 and FX5 images, with none of these having a CCC value greater than 0.70. Interestingly, only two RFs significantly correlated for both the FX5 and dT2 comparisons (GLCM SumAverage and GLSZM GLN). The only difference between FX1 and FX5 images is that they were taken before and after RT, respectively. As previously mentioned, our analysis of FX1 and pMR images shows that there should be correlation between RFs extracted from the same modality. However, since FX1 and FX5 data do not correlate well, we can deduce that most of the difference in RF values is because of the RT that took place between the acquisition of each image. In contrast, the RFs that correlate significantly between FX1 and FX5 are likely not useful for delta-radiomic analysis since they appear to not be affected by RT. Although it is unclear what this change in values means physically, our analysis gives hope for further investigation into this topic.

Results of our comparison between pMR and FX5 images were very similar to those between FX1 and FX5, with eleven RFs correlating significantly. The change in RFs for each of these comparisons was very comparable, with 33 features having their CCC values deviate by less than 0.1. This is an exciting result, as it shows that the change in RFs due to RT intervention is moderately reproducible. This makes sense logically, as pMR and FX1 are images that are both acquired pre-treatment. When comparing each of them to the same post-treatment scan, we would assume that the changes of each would also be very comparable. This is a promising realization for future study, as we can now reliably say that there is a measurable, reproducible signal found in RFs that is caused directly by treatment.

The only feature that did not significantly correlate between pMR and FX1 images was GLSZM Small Zone Low Gray-Level Emphasis (SZLGE). To understand why this feature did not correlate, it was found that this feature emphasized image data that includes small areas of low gray-level pixels. The most logical physical feature of an image that this RF could correspond to would merely be noise. Noise is variable across images taken even on the same machine, so there is an understandable random distribution of noise across different images. Therefore, it makes sense that this feature does not significantly correlate across modalities.

Previous studies have shown the predictive power of extracting RFs through low-Tesla MRI regarding pancreatic cancer (34, 35). Other studies have shown the use of these methods in the treatment of lung cancer (36) and other soft tissue sarcomas (37). There has been little research on the relationship with prostate cancers, which this study aimed to assist in. One study has previously shown that there are significant differences between RFs extracted from different areas of the prostate, but none have shown the relationship between those extracted on different strength magnets. This study demonstrated the value in investigating this relationship, as there are significant and relevant differences between different machines. We have also shown that RFs are repeatable between images taken across a period of days to weeks, while previous studies have only demonstrated repeatability in patient scans separated by a few minutes.

The findings of this study support and verify other research investigating the robustness and repeatability of MR-based radiomic research methods. Several studies have shown comparable results on the repeatability of RFs extracted on low-Tesla machines, but only with the use of phantom measurements (38). This study improves upon the finding of these investigations by demonstrating comparable results in real prostate cancer patients. One study investigated repeatability of features in glioblastoma patients (38), but this study was the first to strictly use prostate cancer patients.

The main takeaways from these results are those relating to the repeatability and reproducibility of RF data. Results show that radiomic data is highly repeatable, meaning that we can obtain data over several instances using the same machine and obtain comparable results. Conversely, it was shown that RF data is not very reproducible, meaning that acquisition of radiomic data on different machines or modalities may not provide helpful insight. Therefore, it is logical that data acquisition in future longitudinal radiomics studies be done on the same modality (onboard CBCT or low-Tesla MRI).

Perhaps the most clinically relevant result in this study was when comparing the change between FX1 and FX5 and the change between pMR and FX5. Our results showed that RT directly resulted in large changes in RF data on the same imaging modality, and that this change is reproducible. The field of delta-radiomics aims to quantitatively predict outcomes of treatment based off changes in RF data extracted over the progression of RT. The results of this study are a proof of concept for the quality of imaging radiomics to be used in delta-radiomic studies, demonstrating validity in this sort of analysis.

Small sample size is a limitation to this study. It is likely that more correlations between modalities would be significant with the addition of extra patients. This study also only examined these correlations on a single machine, future confirmations of the trends shown on other onboard MRI or CT machines could prove to be great support to these results. Our comparison between FX1 and dT2 could also prove to be more robust in future study be only including patients that were imaged on the exact same diagnostic scanner. Even though we narrowed the cohort of this comparison to only include patients on the same brand of scanner, it is still questionable as to whether or not our poor reproducibility was a result of including slightly different machinery. Future radiomics studies comprising of diagnostic MRI and on-board MRI from MRI-LINAC would benefit from keeping variability in machines low.

## 5 Conclusions

This study demonstrated that radiomic features can be reliably repeatable on the same imaging machine integrated with a MR-LINAC. RFs were also shown to be not very reproducible between different types of machinery. However, we did show that there is significant, reproducible change in RF data as treatment progresses as a direct result of irradiation. With the possibility of improved cancer treatment through radiomic analysis, these results provide promising proof of concept that we may continue to pursue research in this field.

## Data Availability

The datasets generated and/or analyzed during the current study are not publicly available due to still being collected as part of an ongoing clinical trial. Requests to access the datasets should be directed to rdelgadillo@med.miami.edu.

## References

[1] Potosky AL Davis WW Hoffman RM Stanford JL Stephenson RA Penson DF. Five-year outcomes after prostatectomy or radiotherapy for prostate cancer: the prostate cancer outcomes study. J Natl Cancer Inst. (2004) 96:1358–67. doi: 10.1093/jnci/djh259 15367568

[2] Siegel RL Miller KD Fuchs HE Jemal A. Cancer statistics, 2021. CA Cancer J Clin. (2021) 71:7–33. doi: 10.3322/caac.21654 33433946

[3] Sung H Ferlay J Siegel RL Laversanne M Soerjomataram I Jemal A. Global cancer statistics 2020: GLOBOCAN estimates of incidence and mortality worldwide for 36 cancers in 185 countries. CA Cancer J Clin. (2021) 71:209–49. doi: 10.3322/caac.21660 33538338

[4] Iorio GC Spieler BO Ricardi U Pra Dal A. The impact of pelvic nodal radiotherapy on hematologic toxicity: A systematic review with focus on leukopenia, lymphopenia and future perspectives in prostate cancer treatment. Crit Rev Oncol Hematol. (2021) 168:103497. doi: 10.1016/j.critrevonc.2021.103497 34666186

[5] Schaeffer E Srinivas S Antonarakis ES Armstrong AJ Bekelman JE Cheng H. NCCN guidelines insights: prostate cancer, version 1.2021. J Natl Compr Canc Netw. (2021) 19:134–43. doi: 10.6004/jnccn.2021.0008 33545689

[6] Zaorsky NG Harrison AS Trabulsi EJ Gomella LG Showalter TN Hurwitz MD. Evolution of advanced technologies in prostate cancer radiotherapy. Nat Rev Urol. (2013) 10:565–79. doi: 10.1038/nrurol.2013.185 24018567

[7] Zaorsky NG Showalter TN Ezzell GA Nguyen PL Assimos DG D'Amico AV. ACR Appropriateness Criteria for external beam radiation therapy treatment planning for clinically localized prostate cancer, part II of II. Adv Radiat Oncol. (2017) 2:437–54. doi: 10.1016/j.adro.2017.03.003 PMC5605284 29114613

[8] Dang A Kupelian PA Cao M Agazaryan N Kishan AU. Image-guided radiotherapy for prostate cancer. Transl Androl Urol. (2018) 7:308–20. doi: 10.21037/tau.2017.12.37 PMC6043755 30050792

[9] Chang YC Ackerstaff E Tschudi Y Jimenez B Foltz W Fisher C. Delineation of tumor habitats based on dynamic contrast enhanced MRI. Sci Rep. (2017) 7:9746. doi: 10.1038/s41598-017-09932-5 28851989 PMC5575347

[10] Delgadillo R Ford JC Abramowitz MC Pra Dal A Pollack A Stoyanova R. The role of radiomics in prostate cancer radiotherapy. Strahlenther Onkol. (2020) 196:900–12. doi: 10.1007/s00066-020-01679-9 PMC7545508 32821953

[11] Peng Y Jiang Y Yang C Brown JB Antic T Sethi I. Quantitative analysis of multiparametric prostate MR images: differentiation between prostate cancer and normal tissue and correlation with Gleason score–a computer-aided diagnosis development study. Radiology. (2013) 267:787–96. doi: 10.1148/radiol.13121454 PMC6940008 23392430

[12] Shiradkar R Podder TK Algohary A Viswanath S Ellis RJ Madabhushi A. Radiomics based targeted radiotherapy planning (Rad-TRaP): a computational framework for prostate cancer treatment planning with MRI. Radiat Oncol. (2016) 11:148. doi: 10.1186/s13014-016-0718-3 27829431 PMC5103611

[13] Stoyanova R Takhar M Tschudi Y Ford JC Solorzano G Erho N. Prostate cancer radiomics and the promise of radiogenomics. Transl Cancer Res. (2016) 5:432–47. doi: 10.21037/tcr.2016.06.20 PMC5703221 29188191

[14] Tanadini-Lang S Bogowicz M Veit-Haibach P Huellner M Pauli C Shukla V. Exploratory radiomics in computed tomography perfusion of prostate cancer. Anticancer Res. (2018) 38:685–90. doi: 10.21873/anticanres.12273 29374691

[15] Vignati A Mazzetti S Giannini V Russo F Bollito E Porpiglia F. Texture features on T2-weighted magnetic resonance imaging: new potential biomarkers for prostate cancer aggressiveness. Phys Med Biol. (2015) 60:2685–701. doi: 10.1088/0031-9155/60/7/2685 25768265

[16] Nardone V Reginelli A Grassi R Boldrini L Vacca G D'Ippolito E. Delta radiomics: a systematic review. Radiol Med. (2021) 126:1571–83. doi: 10.1007/s11547-021-01436-7 34865190

[17] Simpson G Ford JC Llorente R Portelance L Yang F Mellon EA. Impact of quantization algorithm and number of gray level intensities on variability and repeatability of low field strength magnetic resonance image-based radiomics texture features. Phys Med. (2020) 80:209–20. doi: 10.1016/j.ejmp.2020.10.029 33190077

[18] Ford J Dogan N Young L Yang F. Quantitative radiomics: impact of pulse sequence parameter selection on MRI-based textural features of the brain. Contrast Media Mol Imaging 2018. (2018) p:1729071. doi: 10.1155/2018/1729071 PMC6091359 30154684

[19] Yang F Dogan N Stoyanova R Ford JC. Evaluation of radiomic texture feature error due to MRI acquisition and reconstruction: A simulation study utilizing ground truth. Phys Med. (2018) 50:26–36. doi: 10.1016/j.ejmp.2018.05.017 29891091

[20] Sushentsev N Rundo L Blyuss O Nazarenko T Suvorov A Gnanapragasam VJ. Comparative performance of MRI-derived PRECISE scores and delta-radiomics models for the prediction of prostate cancer progression in patients on active surveillance. Eur Radiol. (2022) 32:680–9. doi: 10.1007/s00330-021-08151-x PMC8660717 34255161

[21] Bieri O Scheffler K. Fundamentals of balanced steady state free precession MRI. J Magn Reson Imaging. (2013) 38:2–11. doi: 10.1002/jmri.24163 23633246

[22] Yang F Ford JC Dogan N Padgett KR Breto AL Abramowitz MC. Magnetic resonance imaging (MRI)-based radiomics for prostate cancer radiotherapy. Transl Androl Urol. (2018) 7:445–58. doi: 10.21037/tau.2018.06.05 PMC6043736 30050803

[23] Traverso A Kazmierski M Zhovannik I Welch M Wee L Jaffray D. Machine learning helps identifying volume-confounding effects in radiomics. Phys Med. (2020) 71:24–30. doi: 10.1016/j.ejmp.2020.02.010 32088562

[24] Traverso A Wee L Dekker A Gillies R. Repeatability and reproducibility of radiomic features: A systematic review. Int J Radiat Oncol Biol Phys. (2018) 102:1143–58. doi: 10.1016/j.ijrobp.2018.05.053 PMC6690209 30170872

[25] Vallieres M Freeman CR Skamene SR El Naqa I. A radiomics model from joint FDG-PET and MRI texture features for the prediction of lung metastases in soft-tissue sarcomas of the extremities. Phys Med Biol. (2015) 60:5471–96. doi: 10.1088/0031-9155/60/14/5471 26119045

[26] Zwanenburg A Vallieres M Abdalah MA Aerts H Andrearczyk V Apte A. The image biomarker standardization initiative: standardized quantitative radiomics for high-throughput image-based phenotyping. Radiology. (2020) 295:328–38. doi: 10.1148/radiol.2020191145 PMC7193906 32154773

[27] Delgadillo R Spieler BO Ford JC Kwon D Yang F Studenski M. Repeatability of CBCT radiomic features and their correlation with CT radiomic features for prostate cancer. Med Phys. (2021) 48:2386–99. doi: 10.1002/mp.14787 33598943

[28] Collewet G Strzelecki M Mariette F. Influence of MRI acquisition protocols and image intensity normalization methods on texture classification. Magn Reson Imaging. (2004) 22:81–91. doi: 10.1016/j.mri.2003.09.001 14972397

[29] Lloyd S. Least squares quantization in PCM. IEEE Trans Inf Theory. (1982) 28:129–37. doi: 10.1109/TIT.1982.1056489

[30] Fave X Zhang L Yang J Mackin D Balter P Gomez D. Impact of image preprocessing on the volume dependence and prognostic potential of radiomics features in non-small cell lung cancer. Trans Cancer Res. (2016) 5:349–63. doi: 10.21037/tcr.2016.07.11

[31] Shafiq-Ul-Hassan M Zhang GG Latifi K Ullah G Hunt DC Balagurunathan Y. Intrinsic dependencies of CT radiomic features on voxel size and number of gray levels. Med Phys. (2017) 44:1050–62. doi: 10.1002/mp.12123 PMC5462462 28112418

[32] Lin LIK. A concordance correlation coefficient to evaluate reproducibility. Biometrics. (1989) 45(1):255–68. doi: 10.2307/2532051 2720055

[33] Fave X Mackin D Yang J Zhang J Fried D Balter P. Can radiomics features be reproducibly measured from CBCT images for patients with non-small cell lung cancer? Med Phys. (2015) 42:6784–97. doi: 10.1118/1.4934826 PMC5148115 26632036

[34] Simpson G Spieler B Dogan N Portelance L Mellon EA Kwon D. Predictive value of 0.35 T magnetic resonance imaging radiomic features in stereotactic ablative body radiotherapy of pancreatic cancer: A pilot study. Med Phys. (2020) 47:3682–90. doi: 10.1002/mp.14200 32329904

[35] Tomaszewski MR Latifi K Boyer E Palm RF El Naqa I Moros EG. Delta radiomics analysis of Magnetic Resonance guided radiotherapy imaging data can enable treatment response prediction in pancreatic cancer. Radiat Oncol. (2021) 16:237. doi: 10.1186/s13014-021-01957-5 34911546 PMC8672552

[36] Lacroix M Frouin F Dirand AS Nioche C Orlhac F Bernaudin JF. Correction for magnetic field inhomogeneities and normalization of voxel values are needed to better reveal the potential of MR radiomic features in lung cancer. Front Oncol. (2020) 10:43. doi: 10.3389/fonc.2020.00043 32083003 PMC7006432

[37] Spraker MB Wootton LS Hippe DS Ball KC Peeken JC Macomber MW. MRI radiomic features are independently associated with overall survival in soft tissue sarcoma. Adv Radiat Oncol. (2019) 4:413–21. doi: 10.1016/j.adro.2019.02.003 PMC6460235 31011687

[38] Cattell R Chen S Huang C. Robustness of radiomic features in magnetic resonance imaging: review and a phantom study. Vis Comput Ind BioMed Art. (2019) 2:19. doi: 10.1186/s42492-019-0025-6 32240418 PMC7099536

